# Supplementary material for: A Pilot Study to Assess the Feasibility of Real-Time Teledentistry in Residential Aged Care Facilities
Source: Healthcare (Basel). 2024 Nov 6;12(22):2216. doi: 10.3390/healthcare12222216 (PMC11594027; doi:10.3390/healthcare12222216)
Supplement: Supplementary file 1 [file healthcare-12-02216-s001.zip › healthcare-3231961-supplementary.pdf]

File S1. Oral health assessment tool (OHAT)

Oral Health Assessment Tool (OHAT) for Dental Screening
modified from Kayser-Jones et al. (1995) by Chalmers (2004)

Patient: ______________________ Completed by: ______________________				Date: ____/____/____
Scores: The final score is the sum of scores from the eight categories and can range from 0 (very healthy) to 16 (very unhealthy). While the cumulative score is important in assessing oral health, the score of each item should be considered individually. Symptoms that are underlined require immediate attention. *If any category has a score of 1 or 2, please arrange for the patient to be examined by a dentist.				
**Category**	**0 = healthy**	**1 = changes ***	**2 = unhealthy ***	**Category scores**
Lips	Smooth, pink, moist	Dry, chapped, or red at corners	Swelling or lump, white/red/ulcerated patch; bleeding/ulcerated at corners	
Tongue	Normal, moist, roughness, pink	Patchy, fissured, red, coated	Patch that is red and/or white, ulcerated, swollen	
Gums and tissues	Pink, moist, smooth, no bleeding	Dry, shiny, rough, red, swollen, one ulcer/sore spot under dentures	Swollen, bleeding gums, ulcers, white/red patches, generalized redness or ulcers under dentures	
Saliva	Moist tissues, watery and free-flowing saliva	Dry, sticky tissues, little saliva present	Tissues parched and red, very little/no saliva present, saliva very thick	
Natural teeth Yes/No	No decayed or broken teeth/roots	1-3 decayed or broken teeth/ roots or teeth very worn down	4 or more decayed or broken teeth/roots, or fewer than 4 teeth, or very worn down teeth	
Dentures Yes/No	No broken areas or teeth, dentures regularly worn	1 broken area/ tooth or dentures only worn for 1-2 hrs daily, or loose dentures	More than 1 broken area/tooth, denture missing or not worn, needs denture adhesive	
Oral cleanliness	Clean, no food particles or tartar in mouth or on dentures	Food particles/ tartar/ plaque in 1-2 areas of the mouth or on small area of dentures or bad breath	Food particles/tartar/plaque in most areas of the mouth or on most of dentures or severe halitosis (bad breath)	
Dental pain	No behavioral, verbal, or physical signs of dental pain	Verbal &/or behavioral signs of pain such as pulling at face, chewing lips, not eating, aggression	Physical signs such as facial swelling, sinus on gum, broken teeth, large ulcers, and verbal and/or behavioral signs such as pulling at face, chewing lips, not eating, aggression	
☐ Arrange for patient to be examined by a dentist.<br>☐ Patient or family/guardian refuses dental treatment.<br>☐ Review this patient's oral health again on (date): ___/___/___				TOTAL SCORE: ____ 16

Chalmers J, Johnson V, Tang JH, Titler MG. Evidence-based protocol: oral hygiene care for functionally dependent and cognitively impaired older adults. *J Gerontol Nurs.* 2004 Nov;30(11):5-12.

This tool was based on the work of Chalmers 2005 [14]. This instrument was chosen as it was standard approach used in the two RACFs in the study.

File S2. Background questionnaire

Participant ID: Date

1. What is your age? (in whole years) __________ years
2. What is your gender?
   ☐ Male ☐ Female
3. What ethnicity/ race do you associate with?
   ☐ White/ Caucasian/ Europe ☐ Asian ☐ Pacific Islander
   ☐ Indigenous Australian/ Torres Strait Island ☐ Hispanic/ Latino
   ☐ Other: please specify ________________
4. What is the highest level of education you have completed?
   ☐ High School ☐ Diploma ☐ Bachelor
   ☐ Masters/ Postgraduate ☐ Other: please specify ________________
5. What best describes your current employment status?
   ☐ Employed (Full/ Part time) ☐ Self-employed ☐ Unemployed
   ☐ Cannot work due to disability ☐ Retired
6. Do you currently smoke any tobacco products eg cigarettes, cigars or pipes OR smokeless tobacco products (eg snuff, chewing tobacco, e-cigarettes)?
   ☐ Yes ☐ No
   If "yes"
   Specify which product: ______ Age when you first started _____ years old
   How much do you smoke ________ per day OR _______ per week
7. Do you drink any alcohol?
   ☐ Yes ☐ No
   If "yes" How much do you drink? ________ drinks per day
8. The following questions relate to your <u>current</u> oral health status and related behaviours:
   a. During the past 12 months, did your teeth, gums or mouth cause any pain or discomfort?
      ☐ Yes ☐ No ☐ Unsure
   b. How long has it been since your last visited a dentist or a dental clinic?
      ☐ In the last 12 months ☐ More than 1 year, less than 2 year
      ☐ More than 2 years ☐ Never received dental care
   c. Do you have a regular dentist?
      ☐ Yes ☐ No
   d. Reason for your last dental visit (e.g. check up, tooth pain, consultation)?
      Please specify ____________
   e. How important is your oral health to you?
      ☐ Important ☐ Neutral ☐ Not important
   f. How often do you clean your teeth?
      ☐ Once a day ☐ Twice a day ☐ More than twice a day
      ☐ Once a week ☐ Once a month ☐ Never clean them
   g. Do you use any of the follow to clean your teeth?
      ☐ Toothbrush ☐ Interdental brush ☐ Floss/ flossettes
      ☐ Toothpicks ☐ Others: please specify ________________

   h. Do you use any toothpastes or other cleaning products?
      ☐ Toothpaste with fluoride ☐ Toothpaste without fluoride
      ☐ Denture cleaning products ☐ Others: please specify ________________
   i. Have you ever experienced any of the following barriers getting dental care?
      ☐ Can't get a dental appointment when you need it
      ☐ Can't afford dental care
      ☐ Do not have transportation to dental appointments
      ☐ Dental providers are not accessible

☐ Others: please specify ________________

9. For residents only, before coming into aged care
   a. How often did you see a dentist?
      ☐ In the last 12 months ☐ More than 1 year, less than 2 year
      ☐ More than 2 years ☐ Never received dental care
   b. Did you have a regular dentist?
      ☐ Yes ☐ No
   c. Main reason for dental visit (e.g. check up, tooth pain, consultation)?
      Please specify ____________
10. Has COVID-19 impacted on your ability to visit a dentist?
    ☐ Yes ☐ No ☐ Unsure
11. The following questions relate to telehealth/ teledentistry experience:
    a. Have you participated in telehealth?
       ☐ Yes ☐ No ☐ Unsure

    If "yes", How was it conducted?
       ☐ Telephone ☐ Video ☐ Other, please specify:___________
    b. Have you participated in teledentistry?
       ☐ Yes ☐ No ☐ Unsure

    If "yes", How was it conducted?
       ☐ Telephone ☐ Video ☐ Other, please specify:____________

File S3. Adult oral health assessment form

Date: Participant ID: Examiner:

Dentition status

		18	17	16	15	14	13	12	11	21	22	23	24	25	26	27	28	
Crown	(45)																	60)
Root	(61)																	(76)
Crown	(77)																	(92)
Root	(93)																	(108)
		48	47	46	45	44	43	42	41	31	32	33	34	35	36	37	38	

Permanent teeth

Status

0 = Sound
1 = Caries
2 = Filled w/caries
3 = Filled, no caries
4 = Missing due to caries
5 = Missing for any another reason
6 = Fissure sealant
7 = Fixed dental prosthesis/crown abutment, veneer, implant
8 = Unerupted
9 = Not recorded

Loss of attachment

Severity
0 = 0–3 mm
1 = 4–5 mm Cemento-enamel junction (CEJ) within black band
2 = 6–8 mm CEJ between upper limit of black band and 8.5 mm ring
3 = 9–11 mm CEJ between 8.5 mm and 11.5 mm ring
4 = 12 mm or more CEJ beyond 11.5 mm ring
X = Excluded sextant
9 = Not recorded

* Not recorded under 15 years of age

Index teeth

	17/16	11	26/27	
(173)				(175)
(176)				(178)
	47/46	31	36/37	

Dental erosion

Severity ☐ (180)

0 = No sign of erosion
1 = Enamel lesion
2 = Dentinal lesion
3 = Pulp involvement

Number of teeth affected

(181) ☐☐ (182)

Oral mucosal lesions

☐ (186) ☐ (189)
☐ (187) ☐ (190)
☐ (188) ☐ (191)

Condition

0 = No abnormal condition
1 = Malignant tumour (oral cancer)
2 = Leukoplakia
3 = Lichen planus
4 = Ulceration (aphthous, herpetic, traumatic)
5 = Acute necrotizing ulcerative gingivitis (ANUG)
6 = Candidiasis
7 = Abscess
8 = Other condition (specify if possible)
9 = Not recorded

Location

0 = Vermillion border
1 = Commissures
2 = Lips
3 = Sulci
4 = Buccal mucosa
5 = Floor of the mouth
6 = Tongue
7 = Hard and/or soft palate
8 = Alveolar ridges/gingiva
9 = Not recorded

Denture(s)

Upper ☐ (192) **Lower** ☐ (193)

Status

0 = No denture
1 = Partial denture
2 = Complete denture
9 = Not recorded

Intervention urgency ☐ (194)

0 = No treatment needed
1 = Preventive or routine treatment needed
2 = Prompt treatment (including scaling) needed
3 = Immediate (urgent) treatment needed due to pain or infection of dental and/or oral origin
4 = Referred for comprehensive evaluation or medical/dental treatment (systemic condition)

The form was adapted from the WHO Oral Health Assessment [28].

File S4. Post-study evaluation questionnaire

Post evaluation questionnaire

This is a semi-structure interview and the following is a guideline of the questions that would be asked

1. How does teledentistry exam and consultation compare to face-to-face?
    a. Do you prefer one over the other?
    b. What are somethings you liked or disliked about each method?

2. Did you find it helpful receiving oral hygiene instructions through teledentistry?

3. Can you foresee teledentistry as a way to address unmet oral health

4. For staff at the RACF:
    a. Do you find that this study has helped you understand more about oral health
    b. Do you feel more confident about carrying oral health care for your residents?
    c. Do you have suggestions as to how we can help your team with improving oral health knowledge?

5. Any feedback regarding the study?
    a. Can you please tell me aspects you liked about:
        i. Teledentistry
        ii. Oral hygiene instruction
        iii. In person examination
    b. Can you please give some feedback on what can be improved?

File S5. Oral hygiene care plan (OHCP)

Oral Hygiene Care Plan (Chalmers, 2000)

Resident: ______________________ **Study ID:**______ ***baseline / 3-mth / 6-mth***
Completed by: ______________________ **Date:** __/__/__ ***(please circle)***

Dentist: *public or private* *(please circle)* **Name:** ______________________ **Phone**: __________

**List all dental appointments**:			**Staff to help with oral hygiene care problems**: _______
**Dentures**:	Upper	Full / Partial / Not worn/ No denture/ Named	Attempt denture cleaning: ☐ daily ☐ when possible
(please circle)	Lower	Full / Partial / Not worn/ No denture/ Named	Best time to clean dentures: ____________
**Natural teeth**:	Upper	Yes / No / Roots present	Attempt teeth cleaning: ☐ daily ☐ when possible
(please circle)	Lower	Yes / No / Roots present	Best time to clean teeth : ____________

Types and frequency of assistance needed with oral hygiene care *(please tick all that apply and circle frequency required)*		Regular problems with oral hygiene care : *(please tick all that apply)*	
	☐ no assstance needed		☐ forgets to do oral hygiene care
	☐ reminding / prompting / task breakdown needed		☐ won't open mouth
	☐ supervision/checking of oral hygiene needed		☐ refuses oral hygiene care
	☐ full assistance needed from staff		☐ does not understand
	☐ use brdging / chaining / distraction techniques		☐ is aggressive / kicks / hits
	☐ use electric / suction toothbrush		☐ can't swallow properly
	☐ use backward bent toothbrush for access		☐ can't rinse and spit
	☐ use biteblock		☐ bites tocthbrush and/or staff
	☐ use chlorhexidine spray bottle/gel *daily/weekly*		☐ constantly grinding/chewing
	☐ use fluoride spray bottle/gel *daily/weekly*		☐ head faces downwards
	☐ use Neutrafluor 5000 toothpaste *daily/weekly*		☐ other ____________
	☐ use Oral Balance gel for dry mouth		____________
	☐ other ____________		

This care plan was obtained from Australia Institute of Health and Welfare and was adapted from the work of Chalmers 2000 [9]. This instrument was chosen as it was the standard approach used in the two RACFs in the study.

File S6. Study protocol excerpts

Teledentistry Research - instruction for staff

Dr Candy Fung
Candy.fung@health.qld.gov.au

1 Instruction to set up *Mouthwatch* intra-oral camera

The *Mouthwatch* intra-oral camera is a USB operated device. Please plug it into a USB port in the computer/ laptop.

There is a single-use barrier sleeve that must be used for the camera for each resident/ staff. Please insert the camera into the packet as shown. This will ensure that the camera is facing the clear window in the barrier sleeve

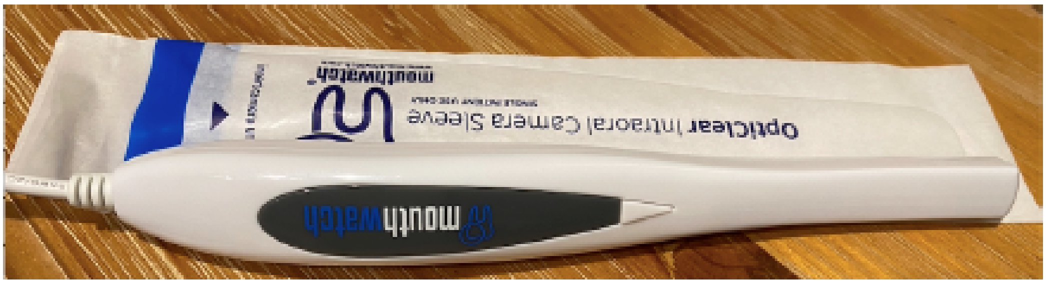

To take videos/ photos, use the camera like you would use a toothbrush. Please give the resident and yourself lots of breaks in between to have a rest. I am happy to guide you through as I will be connected through a video chat. Important thing is to have a play with the camera and it is quite intuitive.

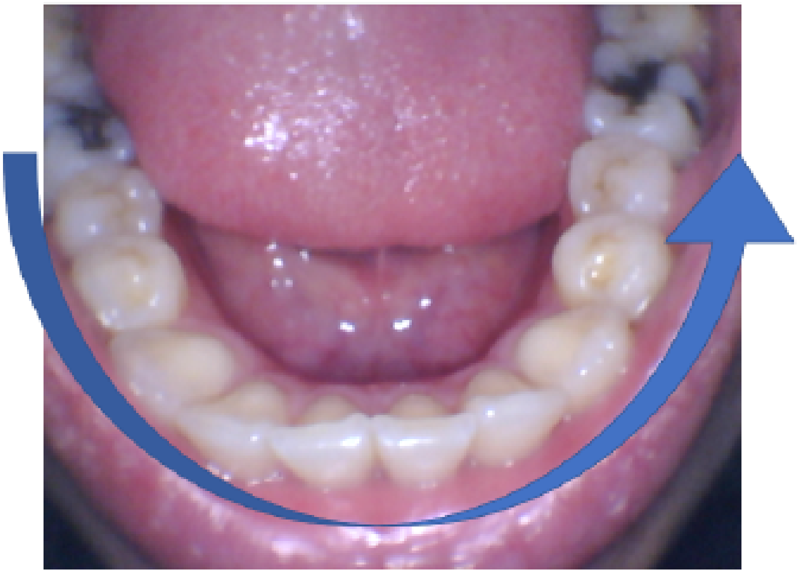

Example of using the camera to video the bottom teeth from right to left on the cheek side of the bottom teeth

2 Instructions for Teams meeting

Login to ***Microsoft Teams*** through desktop or the search bar:

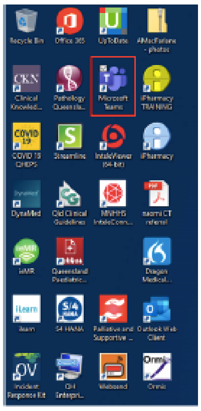

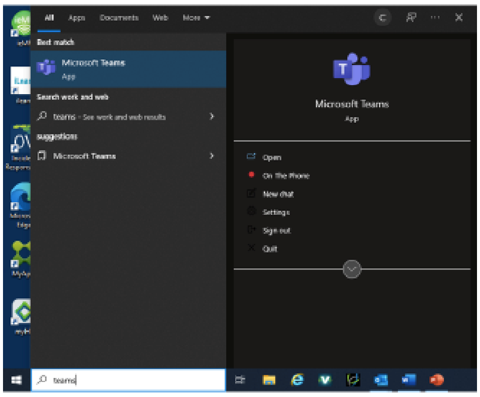

This would open up once you are in a meeting:

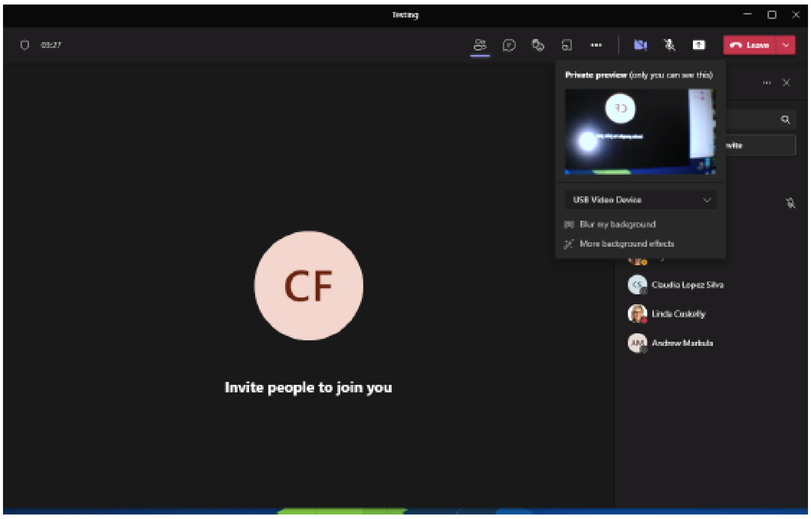

Hover your mouse at this icon 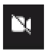

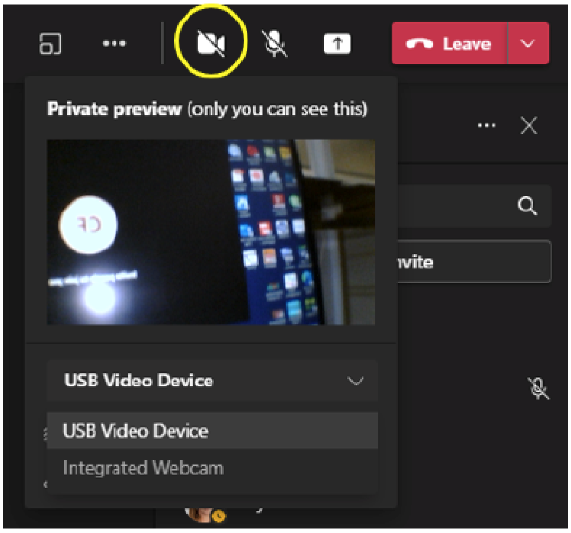

This will allow you to switch between the Webcam on your computer or

USB Video Device – for the *Mouthwatch* intra-oral camera

You can switch freely between the two depending on when we are talking to the resident (webcam) or looking inside the mouth (*Mouthwatch* intra-oral camera)

Table S1. OHAT raw scores.

OHAT parameters	Overall (N)	Healthy (0)		Changed (1)		Unhealthy (2)	
		N	%	N	%	N	%
**Lips**							
Teledentistry	24	14	58.3	9	37.5	1	4.2
Exam	24	9	56.3	5	31.3	2	12.5
Nurse (baseline)	16	16	100	0	0	0	0
**Tongue**							
Teledentistry	24	11	45.8	13	54.2	0	0
Exam	24	12	75	4	25	0	0
Nurse (baseline)	16	16	100	0	0	0	0
**Gums and Tissues**							
Teledentistry	24	16	66.7	6	25	2	8.3
Exam	24	7	43.8	4	25	5	31.3
Nurse (baseline)	16	16	100	0	0	0	0
**Saliva**							
Teledentistry	24	19	79.2	4	16.7	1	4.2
Exam	24	12	75	3	18.8	1	6.3
Nurse (baseline)	16	16	100	0	0	0	0
**Natural teeth**							
Teledentistry	19	7	36.8	4	21.1	8	42.1
Exam	19	6	31.6	5	26.3	8	42.1
Nurse (baseline)	12	4	33.3	3	25	5	41.7
**Dentures**							
Teledentistry	9	3	33.3	1	11.1	5	55.6
Exam	9	4	44.4	1	11.1	4	44.4
Nurse (baseline)	6	4	66.7	1	16.7	1	16.7
**Oral Cleanliness**							
Teledentistry	24	9	37.5	10	41.7	5	20.8
Exam	24	9	37.5	9	37.5	6	25
Nurse (baseline)	16	11	68.8	5	31.3	0	0
**Dental pain**							
Teledentistry	24	17	70.8	6	25	1	4.2
Exam	24	20	83.3	3	12.5	1	4.2
Nurse (baseline)	16	15	93.8	1	6.3	0	0

This table shows data collected using (1) OHAT score from the teledentistry assessment, (2) OHAT score based on the WHO examination sheet, and (3) OHAT scores from the medical chart, as scored by nurses as a baseline score for residents on admission.
